# Non-Covalent Interactions Between Quercetin and Rice Bran Protein: Mechanisms and Functional Properties

**DOI:** 10.3390/foods14172923

**Published:** 2025-08-22

**Authors:** Shirang Wang, Dianyu Yu, Tengyu Wang, Liping Zhou, Xu Han

**Affiliations:** 1 Institute of Natural Resources and Ecology, Heilongjiang Academy of Sciences, Harbin 150040, China; wangshirang20230076@hrbu.edu.cn; 2 School of Food Engineering, Harbin University, Harbin 150086, China; 3 College of Food Science, Northeast Agricultural University, Harbin 150030, China; dyyu2000@126.com (D.Y.); wangtengyu2017@126.com (T.W.)

**Keywords:** plant polyphenols, rice bran protein-quercetin complex, antioxidant activities, foaming properties, emulsifying properties

## Abstract

Rice bran protein (RBP) is an important plant protein, but its functional properties are reduced due to the presence of disulfide bonds in the structure. Polyphenol modification is an effective strategy to improve protein functional properties. However, the interactions between quercetin (Que) and RBP have not been well-studied. In this study, we explored the mechanism of non-covalent interactions between RBP and Que and systematically evaluated the improvement of functional properties of the RBP–Que complex. The results revealed that the addition of Que can significantly affect the particle size, ζ-potential and protein flexibility of the RBP–Que complex, and the non-covalent interactions significantly altered the secondary structure (α-helix content decreased to 20.28%, β-sheet decreased to 22.02%, β-turn increased to 29.30% and random coil increased to 28.40%) and the tertiary conformation of RBP. Spectroscopic data showed that static quenching occurred. Thermodynamic parameters showed that ΔG, ΔH, and ΔS were negative, revealing that the binding process was spontaneous and exothermic and the main reactive bonds were the hydrogen bond and the van der Waals force. When the Que concentration was 120 μmol/g, the emulsifying and foaming properties were improved by 57.72% and 71.88% compared with the RBP, respectively. The study will expand the application of RBP in the food and beverage processing industry.

## 1. Introduction

Rice bran is an excellent nutritional source of bioactive compounds, including phytochemicals such as ferulic acid, γ-oryzanol, γ-aminobutyric acid, and phytosterols; it also contains 12–16% protein [1,2]. Rice bran protein (RBP) is considered to be a high-quality plant protein extracted from rice bran. It has been demonstrated to have clear application value in infant formula food and in food for special medical purposes due to its hypoallergenicity and well-balanced amino acid composition [3,4]. However, as disulfide bonds exist in the structure of RBP, the solubility and emulsifying properties are poor; thus, the application of RBP in the food industry is limited [5,6]. With the growing demand for functionalized plant proteins, researchers have developed various modifications to break through this bottleneck. Researchers have improved the functional properties of RBP by means of glycosylation and phosphorylation. For example, Luo et al. [7] grafted glucose on RBP using the Maillard reaction and found that the emulsifying properties were significantly increased after a glycosylation reaction. Hu et al. [8] modified RBP by phosphorylation to improve its solubility and emulsifying properties. However, these modifications may be accompanied by the generation of by-products and require relatively harsh reaction conditions.

Given the constraints of the structural properties of RBP, scholars have begun to explore ways to enhance the functional properties through molecular modification with small molecule compounds [9]. Plant polyphenols have antioxidant, anti-inflammatory, and other functions. Novel complexes can be formed by the interactions between polyphenols and proteins and the functionality of proteins can be improved effectively [10]. Generally speaking, the interactions of proteins with polyphenols fall mainly into two modes: non-covalent and covalent interactions [11]. Non-covalent interactions have attracted much attention due to the simplicity of the operation and the mild conditions required [12]. It was shown that chlorogenic acid improved the functional properties of the complexes, through non-covalent interactions with soybean protein [13], and Wang et al. [14] reported that the non-covalent binding of tannic acid to hemp seed protein was utilized to improve the emulsifying properties. Quercetin (Que) is a flavonoid polyphenol widely found in fruits, vegetables, and grains, with biological activities such as antioxidant and antibacterial properties [15]. However, Que has very low water solubility and limited bioavailability, and there is an urgent need to improve its physicochemical properties through carrier systems [16]. Ke et al. [17] and Cheng et al. [18] investigated the interactions of casein and whey protein concentrate with quercetin, respectively, revealing that Que could improve the functionalities of the two proteins. Therefore, we hypothesized that the combination of RBP and Que could improve the functional properties of RBP to expand its applications.

To verify our hypothesis, RBP–Que complexes were prepared with RBP and different concentrations of Que via non-covalent interactions. The physicochemical properties, structure changes, and functionality of the complexes were analyzed comprehensively. The effects of this binding method on the structure of RBP were investigated. The non-covalent reaction mechanism between RBP and Que was clarified. The influence of structural changes on the functional properties of the protein was evaluated. This study should promote the comprehensive utilization of rice processing by-products and functional food innovation.

## 2. Materials and Methods

### 2.1. Materials

Fresh rice bran was provided from Heilongjiang Great Northern Wilderness Agribusiness Group Co., Ltd. (Harbin, Heilongjiang, China). The RBP (purity 90.63%, dry basis, as determined by the Kjeldahl method) used in this study was self-extracted by our laboratory. Quercetin (purity 97%) was purchased from Macklin Co., Ltd (Shanghai, China). The 8-Anilino-1-naphthalenesulfonic acid (ANS) and sodium dodecyl sulfate (SDS) were supplied by Solarbio Science & Technology Co., Ltd. (Beijing, China). 1,1-diphenyl-2-picrylhydrazyl (DPPH) and 2,2′-azino-bis (3-ethylbenzothiazoline-6-sulfonic acid) (ABTS) were provided by Sigma-Aldrich (Saint Louis, MO, USA). All chemicals were of analytical grade.

### 2.2. Preparation of RBP and RBP–Que Complexes

#### 2.2.1. Extraction of RBP

The rice was harvested in the autumn. Rice bran was collected immediately after production and transported in a cold chain to extract the RBP. The RBP used in this study was self-extracted by our laboratory; the detailed extraction method was as follows: Fresh rice bran was defatted with 10 volumes of N-hexane for 4 h and air-dried overnight at 25 °C. The defatted rice bran was mixed with 10-fold deionized water, and the pH value was adjusted to 9.0 with 2 mol/L NaOH. After stirring for 2 h using a magnetic stirrer (JB-2, Lei Ci Xin Jing Instrument Co., Ltd., Shanghai, China), the mixing suspension was centrifuged at 7500 rpm for 20 min using a GL-21M refrigerated centrifuge (Xiangyi Centrifuge Instrument Co., Ltd., Changsha, China) at 4 °C to remove the insoluble material. Then, the supernatant was adjusted to pH 4.5 with 2 mol/L HCl. The precipitated protein was obtained by centrifugation at 4000 rpm for 5 min at 4 °C. After washing with 5-fold deionized water twice, the protein was centrifugated at 4000 rpm for 5 min, and the pH value was adjusted to 7.0 with 2 mol/L NaOH. Finally, the RBP solution was freeze-dried using a freeze dryer (FD-2A, Biocool, Beijing, China) at −50 °C and stored at −20 °C.

#### 2.2.2. Preparation of RBP–Que Complexes

According to the method proposed by Wang et al. [12], some modifications were made to prepare the complexes. A series of concentrations of Que (in DMSO) were prepared and mixed with RBP solution to make the complexes. The RBP concentration was 10 mg/mL in the mixture and the Que concentrations were 15, 30, 60, 120, and 240 μmol/g protein, respectively. The mixtures were adjusted to pH 7.0 with 2 mol/L NaOH and stirred in the dark for 2 h using a magnetic stirrer (JB-2, Lei Ci Xin Jing Instrument Co., Ltd., Shanghai, China) to obtain the RBP–Que complexes. The complexes were dialyzed in a 3000 Da dialysis bag for 48 h. The samples of the complexes were named R-Q15, R-Q30, R-Q60, R-Q120, and R-Q240. All the samples were freeze-dried using a freeze dryer (FD-2A, Biocool, Beijing, China) at −50 °C and stored at −20 °C until use.

### 2.3. Molecular Characteristics

#### 2.3.1. Quercetin Binding Ratio

The determination for the total phenolic content was carried out using the Folin–Ciocalteu method [19]. The RBP–Que complex solution was dialyzed using a 3000 Da dialysis bag, then the dialyzed solution was used to analyze the total phenolic content (polyphenols not bound to RBP). The quercetin binding ratio was calculated using the following equation:

(1) $$Quercetin\;\mathrm{binding}\;\mathrm{ratio}\;\left(\%\right)\;=\;\frac{M_{1}-M_{2}}{M_{1}}\;\times \;100$$

where *M*_1_ is the amount of Que added (g) and *M*_2_ is the amount of Que in dialyzed solution (g).

#### 2.3.2. Particle Size, ζ-Potential and Polydispersity Index (PDI)

The samples were diluted with PBS (0.01 mol/L, pH 7.0) to a protein concentration of 1 mg/mL. A Zetasizer Nano ZS 90 (Malvern Instrument Ltd., Malvern, Worcestershire, UK) was used to measure the particle size, ζ-potential and PDI of the RBP–Que complexes. Samples were placed in equilibrium for 2 min before the test. The experimental conditions were 25 ± 0.1 °C. Each sample was measured 3 times and the average value was taken.

#### 2.3.3. Turbidity

The sample was diluted with PBS (0.01 mol/L, pH 7.0) to 3 mg/mL. The absorbance value at 600 nm was measured as turbidity using a UV spectrophotometer (UV1901, Lengguang Technology Co., Ltd., Shanghai, China). The cuvettes were disposable.

#### 2.3.4. Molecular Flexibility

The determination of molecular flexibility was conducted following the method of Ma et al. [13] with slight modifications. The 4 mL of sample solution (1 mg/mL) was mixed with 250 μL of trypsin solution (1 mg/mL), and the mixture was thoroughly mixed and incubated in a water bath (HWS26, Yiheng Scientific Instruments Co., Ltd., Shanghai, China) at 38 °C for 5 min. Then, 4 mL of trichloroacetic acid (5.0%) was added. The mixture was centrifuged at 4000 rpm for 30 min. The supernatant was collected and the absorbance was measured at 280 nm.

### 2.4. Structural Characteristics

#### 2.4.1. Fourier Transform Infrared (FTIR) Spectrometry

The freeze-dried RBP–Que complex was mixed with potassium bromide to 1:100. The pressed mixture was scanned with an FTIR spectrometer (Spectrum 3, PerkinElmer, Pontyclun, UK). The scanning conditions were a wavelength range of 500 to 4000 cm^−1^, a scanning frequency of 32, and a resolution of 4 cm^−1^. Baseline correction and Gaussian fitting were performed on the spectra of amide I bands (1600–1700 cm^−1^) using PeakFit 4.12 software to calculate the relative content of each secondary structural content.

#### 2.4.2. Surface Hydrophobicity (H_0_)

The determination of H_0_ followed the method of Liu et al. [20] and used ANS as a fluorescent probe. The specific steps are as follows: Dilute the RBP–Que complex sample with PBS (0.01 mol/L, pH 7.0) to a series of gradient solutions with a protein concentration of 0.0125~0.1 mg/mL. Take 4 mL of the diluted sample solution, add 20 μL of the ANS solution with a concentration of 8 mmol/L, mix thoroughly, and react in the dark for 3 min. Measure the fluorescence intensity using a fluorescence spectrophotometer (F7000, Hitachi, Tokyo, Japan) at an excitation wavelength of 390 nm and an emission wavelength of 470 nm. Draw a standard curve with protein concentration as the horizontal axis and fluorescence intensity as the vertical axis and calculate the initial slope through a linear regression equation. The obtained slope value is H_0_.

#### 2.4.3. Fluorescence Spectrometry

The method of Wang et al. [11] was used to measure the fluorescence spectrum. The RBP–Que complex samples were diluted to 10 mg/mL with a PBS buffer, and then measured using a fluorescence spectrophotometer (F7000, Hitachi, Tokyo, Japan). The excitation wavelength was set at 290 nm, and the emission wavelength scanning range was 300–450 nm. Both the excitation and emission slit widths were set at 2.5 nm.

#### 2.4.4. Fluorescence Quenching Mechanism

The fluorescence intensity of RBP–Que was measured at three temperature conditions of 298.15, 306.15, and 314.15 K to investigate the fluorescence quenching mechanism and thermodynamic parameters, following the method of Ma et al. [13]. After incubating the sample solution (0.2 mg/mL) in a constant-temperature water bath (HWS26, Yiheng Scientific Instruments Co., Ltd., Shanghai, China) for 5 min, spectral scanning was performed using a fluorescence spectrophotometer (F7000, Hitachi, Tokyo, Japan). The excitation wavelength was set to 290 nm, the excitation and emission slit widths were both 5 nm, the scanning speed was 600 nm/min, and the emission wavelength scanning range was 300–500 nm.

### 2.5. Functional Characteristics

#### 2.5.1. Solubility

The determination of protein solubility was carried out by referring to the method proposed by Wang et al. [12]. RBP–Que complex samples were prepared in a solution (10 mg/mL) with pH 7.0. After magnetic stirring for 2 h using a magnetic stirrer (JB-2, Lei Ci Xin Jing Instrument Co., Ltd., Shanghai, China), the solution was centrifuged at 10,000× *g* for 20 min. The protein content in supernatant was measured by the Lowry method [21].

#### 2.5.2. Antioxidant Activities

1. DPPH radical scavenging assay

DPPH radical scavenging rates were analyzed following the method of Blois [22]. Briefly, 2 mL of the sample solution was mixed with 2 mL of DPPH solution (0.2 mM in ethanol) in a dark environment. After incubating for 60 min at room temperature, the absorbance of the mixture was measured at 517 nm using a spectrophotometer (UV1901, Lengguang Technology Co., Ltd., Shanghai, China). The DPPH radical scavenging activity was calculated using the following equation:

(2) $$DPPH\;radical\;scavenging\;activity\;\left(\%\right)=\frac{A_{1}-{\;A}_{2}}{A_{1}}\;\times \;100\%$$

where A_1_ and A_2_ are the absorbance of DPPH solution after adding sample solution and without adding sample solution, respectively.

2. ABTS radical scavenging assay

ABTS radical scavenging rates were evaluated based on the protocol outlined by Siddhuraju [23]. Initially, the ABTS working solution was diluted with ethanol to achieve an absorbance of 0.70 ± 0.02 at 734 nm, which was verified using a spectrophotometer (UV1901, Lengguang Technology Co., Ltd., Shanghai, China). For the assay, 1 mL of the RBP–Que complex solution (0.5 mg/mL) was mixed thoroughly with 3 mL of the pre-adjusted ABTS solution. The mixture was then allowed to stand at room temperature for 1 h in the dark to ensure complete reaction. After incubation, the absorbance of the resulting solution was measured at 734 nm.

The ABTS radical scavenging activity was calculated using the formula:

(3) $$ABTS\;radical\;scavenging\;activity\;\left(\%\right)\;=\;\frac{A_{1}-{\;A}_{2}}{A_{1}}\;\times \;100\%$$

where A_1_ and A_2_ are the absorbance of ABTS solution after adding sample solution and without adding sample solution, respectively.

#### 2.5.3. Foaming Properties

The foaming capacity (FC) and foaming stability (FS) were measured by Wang et al. [12]. Briefly, 30 mL of RBP–Que complex solution was homogenized at 10,000 rpm for 1 min using a high-speed homogenizer (T18 digital Ultra-Turaxx, IKA, Staufen, Germany) to generate foam. Immediately after homogenization, the entire mixture (foam + liquid) was transferred to a 100 mL graduated cylinder, and the total volume was recorded to calculate the FC. The cylinder was then left undisturbed at room temperature, and the residual foam volume was measured after 30 min to determine the FS.

The formulas used for calculations were as follows:

(4) $$\mathrm{FC}\;\left(\%\right)\;=\;\frac{V_{2}\;-V_{1}}{V_{1}}\;\times \;100$$

(5) $$\mathrm{FS}\;\left(\%\right)=\frac{V_{3}-V_{1}}{V_{2}-V_{1}}\;\times \;100$$

where V_1_ (mL) is volume before whipping, V_2_ (mL) is volume after whipping, and V_3_ (mL) is volume after standing.

#### 2.5.4. Emulsifying Properties

The emulsifying properties were determined as described by Wang et al. [11]. The RBP–Que complex solution was diluted with PBS (0.01 mol/L, pH 7.0) to a final protein concentration of 0.2% (*w*/*v*). Soybean oil and the diluted complex solution were mixed at a volume ratio of 1:3, and the mixture was homogenized at 10,000 rpm for 1 min using a high-speed homogenizer (T18 digital Ultra-Turaxx, IKA, Staufen, Germany) to form an emulsion. For emulsifying activity index (EAI) and emulsifying stability index (ESI) determination, 100 μL of the emulsion was pipetted from the bottom of the freshly prepared emulsion immediately (0 min) and 10 min after homogenization, respectively. Each aliquot was added to 9.9 mL of 0.1% SDS solution, vortexed thoroughly to ensure uniform mixing, and the absorbance was measured at 500 nm.

The EAI and ESI were calculated using the following formulas:

(6) $$\mathrm{EAI}\;\left(m^{2}/g\right)\;=\;\frac{2\;\times \;2.303\;\times \;A_{0}\;\times \;100}{1000\;\times \;0.25\;\times \;1\;\times \;0.005}$$

(7) $$\mathrm{ESI}\;\left(\min\right)=\frac{A_{0}}{A_{0}-A_{10}}\;\times \;(T_{10}-T_{0})$$

where A_0_ and A_10_ are the absorbance determined at 0 min and 10 min, respectively. T_0_ is 0 min, and T_10_ is 10 min.

### 2.6. Statistical Analysis

The experiments were performed in triplicate, and the results were expressed as mean ± standard deviation. The SPSS Statistics software (version 22.0, IBM Corp., New York, NY, USA) was used to perform data analysis. Significant differences were assessed by ANOVA followed by Duncan’s multiple comparison test (*p* < 0.05).

## 3. Results and Discussion

### 3.1. Molecular Characteristics

#### 3.1.1. Quercetin Binding Ratio

**Figure 1:**
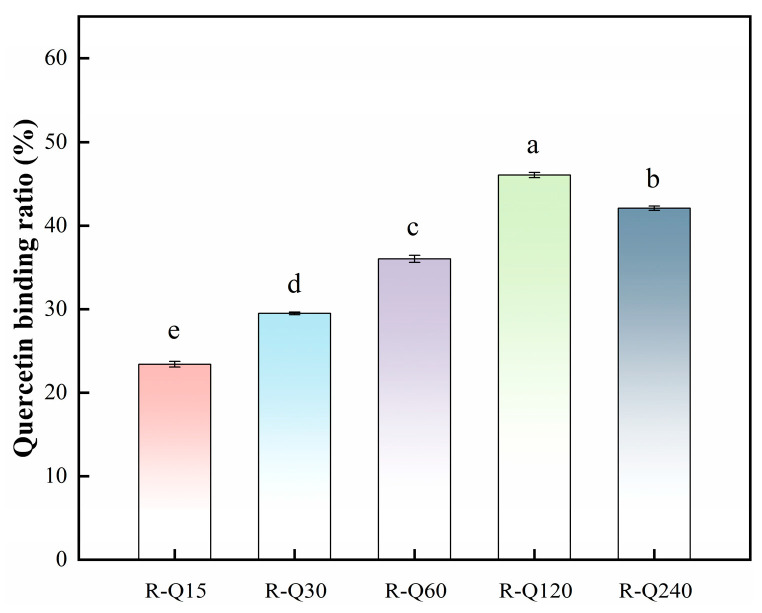
Quercetin binding ratio of rice bran protein (RBP)–quercetin (Que) non-covalent complexes. R-Q15, R-Q30, R-Q60, R-Q120, and R-Q240 represent RBP–Que complexes with different Que concentrations (15, 30, 60, 120, and 240 μmol/g protein, respectively). The error bars indicate the standard deviation (±SD) obtained from triplicate determinations (*n* = 3). Lowercase (a–e) letters indicate significant differences in the quercetin binding ratio of RBP–Que complexes (*p* < 0.05).

The quercetin binding ratio was used to characterize the extent of Que bound to RBP by non-covalent interactions. The experimental results are shown in Figure 1. The results showed that the Que binding ratio increased significantly as the Que concentration increased, but decreased slightly at R-Q240. The interactions between proteins and polyphenols are related to the quantity and position of phenolic hydroxyl groups [24]. The active phenolic hydroxyl groups on the structure of the quercetin molecule were able to cross-link to the RBP by non-covalent interactions. This can unfold the structure of RBP, effectively exposing the hidden binding sites, which can promote the binding ratio of RBP and Que [10]. The electrostatic complementary interactions between the phenolic hydroxyl groups of polyphenols and the side chain groups of RBP were enhanced with the increasing Que concentration. That would drive the non-covalent interactions process further [25]. However, it was found that the Que binding ratio of R-Q240 sample decreased due to the fact that the Que bound to the RBP had already been saturated. Our finding was consistent with Wang et al. [12] and Hao et al. [26].

#### 3.1.2. Particle Size and PDI

**Figure 2:**
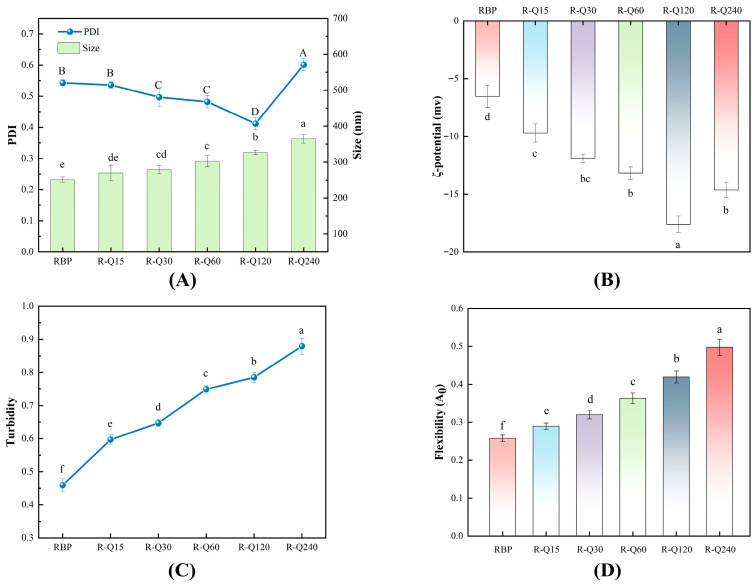
Particle size and PDI (**A**), ζ-potential (**B**), turbidity (**C**), and flexibility (**D**) of rice bran protein (RBP) and RBP–Quercetin (Que) non-covalent complexes. R-Q15, R-Q30, R-Q60, R-Q120, and R-Q240 represent RBP–Que complexes with different Que concentrations (15, 30, 60, 120, and 240 μmol/g protein, respectively). The error bars indicate the standard deviation (±SD) obtained from triplicate determinations (*n* = 3). Uppercase (A–D) and lowercase (a–f) letters indicate significant differences in the PDI, size, ζ-potential, turbidity, and flexibility of RBP and RBP–Que complexes, respectively (*p* < 0.05).

As Figure 2A shows, the average particle sizes of the RBP–Que non-covalent complexes increased significantly compared with the RBP sample. This change in particle size was a result of the enhanced non-covalent interactions between Que and RBP, which led to an increase in the size of the complexes [12]. This finding was in accordance with the study of Sui et al. [27] regarding the non-interactions between soy protein and anthocyanins. The PDI values of the complexes showed a trend of decreasing and then increasing as the Que concentration increased. The PDI gradually decreased from 0.543 to 0.402 in the Que concentration range of 0–120 μmol/g, and then increased to 0.601 at 240 μmol/g. The PDI values decreased gradually with the increase in Que concentration compared to the RBP sample. This was attributed to the non-covalent interactions, which made the protein particles more uniform and more homogeneous. At the same time, moderate electrostatic repulsion can also lead to this phenomenon. The PDI value of the R-Q120 sample was the lowest (*p* < 0.05). This was due to the interactions between RBP and Que at the optimal concentration, which generated more homogeneous protein particles, better protein dispersion, and a more stable solution. The PDI value was significantly larger (*p* < 0.05) for the R-Q240 sample. This was caused by the aggregation of the RBP–Que complex at a higher Que concentration, which resulted in an increase in PDI [28].

#### 3.1.3. ζ-Potential

The ζ-potential of the complex is a key parameter to characterize their surface charge density. Generally speaking, the higher the absolute value indicates, the stronger inter-particle electrostatic repulsion is, and this directly affects the dispersion stability of protein. As Figure 2B shows, the absolute ζ-potential of the RBP–Que complex increased significantly (*p* < 0.05) compared with the RBP sample, with a trend of increasing firstly and then decreasing. The R-Q120 sample gained the highest absolute ζ-potential (16.6 mV). This indicated that the RBP molecule was moderately unfolded, the charged groups were fully exposed, the surface charge distribution was more homogeneous and denser, and the inter-particle electrostatic repulsive force was enhanced, which could effectively inhibit the aggregation of the complex particles [14]. However, the ζ-potential of the R-Q240 sample decreased, which was caused by the aggregation of the complex. The process led to the charged groups being sealed inside the aggregates, the surface charge density decreasing, and the electrostatic repulsion weakening, which had negative effects on the solution system [18]. This was consistent with the trend of particle size and PDI in this study. It can be concluded from the data that the non-covalent interactions can significantly change the electrostatic properties of the protein-polyphenol system. Other researchers’ studies on non-covalent interactions between lactoferrin and theaflavin are consistent with our research findings [29].

#### 3.1.4. Turbidity

Turbidity is an indicator to characterize the aggregation behavior of protein-polyphenol complexes, as it reflects the dynamic changes of the complex intuitively [30]. It can be seen from Figure 2C that the turbidities of the RBP–Que complexes show a continuous increasing trend with the increase in the Que concentration, compared with the RBP sample. This phenomenon may be due to the non-covalent interactions. The assembly of the complex was promoted with the Que concentration increasing, and the particle size of the complexes gradually became larger. The turbidity of the complex was related to its particle size [12]. When the Que concentration reached a critical value, the increase in turbidity was particularly significant, indicating that the micro-aggregation phenomenon in the system had occurred. The research results of Dai et al. [31] confirm that the non-covalent interactions between catechin and soy protein can lead to an increase in turbidity. The results of the present study were consistent with these findings.

#### 3.1.5. Flexibility

Protein flexibility reflects the ability of proteins to rearrange their structure after being affected by changes in the external environment, which is mainly affected by non-covalent and covalent bonds [32]. It directly affect the functional properties of protein. The flexibility results of the RBP and the RBP–Que complexes are as Figure 2D shows. The complex flexibility was significantly increased (*p* < 0.05) as the Que concentration increased. This confirmed that the non-covalent binding mode of Que to RBP could enhance the RBP molecular flexibility significantly. The addition of Que could alter the structure of the RBP, which shifted from a rigid to flexible structure, increasing the molecular flexibility. This was confirmed by the FTIR study in Section 3.2.1. Flexibility can affect the distribution and adsorption capacity of protein molecules on the interface, which has positive effects on solubility and emulsifying properties [33]. This observation was in keeping with Wang et al. [34].

### 3.2. Structural Characteristics

#### 3.2.1. FTIR Spectra

**Figure 3:**
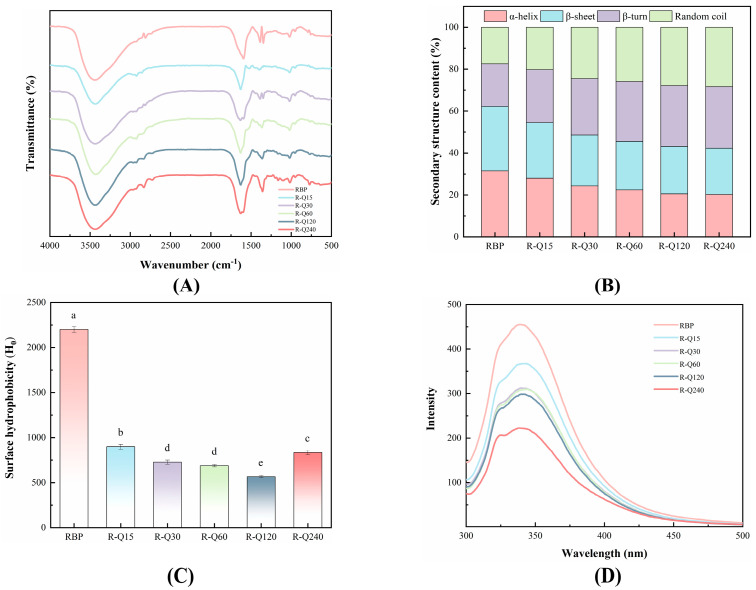
FTIR spectra (**A**), secondary structure content (**B**), surface hydrophobicity (H_0_) (**C**), and fluorescence spectra (**D**) of rice bran protein (RBP) and RBP–Quercetin (Que) non-covalent complexes. R-Q15, R-Q30, R-Q60, R-Q120, and R-Q240 represent RBP–Que complexes with different Que concentrations (15, 30, 60, 120, and 240 μmol/g protein, respectively). The error bars indicate the standard deviation (±SD) obtained from triplicate determinations (*n* = 3). Lowercase (a–e) letters indicate significant differences in the surface hydrophobicity (H_0_) of RBP and RBP–Que complexes, respectively (*p* < 0.05).

The FTIR is sensitive to tracking changes in the characteristic absorption peaks of protein. It is an effective tool to reveal the mechanisms of reactions between protein and polyphenol. Figure 3A shows the FTIR spectra of RBP and RBP–Que. The typical O-H/N-H stretching vibration absorption peak of RBP around 3440 cm^−1^ was changed by the addition of Que. This suggested that the hydrogen bonding was formed during the non-covalent interactions between Que and RBP. In the course of the reaction, the hydrogen bond was formed between the amide group of RBP and the phenolic hydroxyl group of Que. The vibrational frequency of O-H/N-H reduced because of the hydrogen bond, which led the characteristic peaks to shift to lower wave numbers [35]. The peaks near 2960 and 2930 cm^−1^ were mainly C-H stretching vibrations of CH_3_ and CH_2_ groups of the protein side chains. Compared to the RBP, the peaks of the RBP–Que complex in the region were significantly changed. This change may imply that hydrophobic interactions also occurred during the complex formation, meaning that the hydrophobic microenvironment of the RBP was altered due to the Que binding [36,37]. The study of Huang et al. [38] indicated that amino acids such as glutamic acid, arginine and phenylalanine in rice gluten can form complexes with quercetin through non-covalent bonds. The amide I band (1600–1700 cm^−1^) and amide II band (1500–1600 cm^−1^) of protein are highly sensitive to changes in secondary structure. In the Figure 3A, changes were observed in the position of the absorption peaks. It was directly confirmed that the binding of Que interfered with the local environment of the peptide bonds (C=O, C-N, N-H) in the RBP; thus, the structure of RBP was changed [18].

The secondary structure content of the RBP was calculated by Gaussian fitting of the amide I band profile, and the specific information is shown in Figure 3B. It can be seen that α-helix = 31.49%, β-sheet = 30.79%, β-turn = 20.22%, and random coil = 17.50%. The RBP structure was changed by the non-covalent interactions. As the Que addition increased, the α-helix decreased from 31.49% to 20.28%, the β-sheet decreased from 30.79% to 22.02%, the β-turn increased from 20.22% to 29.30%, and the random coil increased from 17.50% to 28.40%. This revealed that the RBP structures were partially unfolded and rearranged, and transformed to a more flexible and stretched state [26]. In general, α-helix and β-sheet represent the rigid structure of protein, and β-turn and random coil represent the flexible structure of protein. The flexibility of the RBP increased as the Que bound with the RBP. This was helpful to the improvement of the functional properties.

#### 3.2.2. Surface Hydrophobicity

Surface hydrophobicity, H_0_, is an indicator reflecting how hydrophobic groups change within protein structures. The H_0_ of the non-covalent complex is shown in Figure 3C. Compared with the RBP sample, the H_0_ of the RBP–Que complex decreased significantly (*p* < 0.05) and showed a decreasing trend as the Que concentration increased. The Que molecules were directly bound with the hydrophobic sites of the RBP by hydrophobic interaction, the hydrophobic sites on the surface of the RBP were shielded, and then the effective binding with the ANS probe was hindered [39]. The exposures of the hydrophobic groups were reduced further with the RBP–Que complex formation. In addition, the Que binding with the RBP may have also caused the hydrophilic groups of the RBP to be exposed, which led to the reduction in H_0_ [40]. The interfacial behavior of the RBP–Que complexes can be profoundly affected by the H_0_ decreasing, which has positive effects on the functional properties. This was consistent with the change pattern between casein and Que on H_0_ [17].

#### 3.2.3. Fluorescence Spectra

Fluorescence spectroscopy is used to monitor the tertiary structure of proteins, which mainly reflects the microenvironmental changes through the fluorescence properties of tryptophan residues. As Figure 3D depicted, the fluorescence intensity of the RBP–Que complex reduced significantly compared with that of the RBP, indicating that the Que exerted an obvious fluorescence quenching effect on the RBP. This quenching phenomenon suggested that as the non-covalent interactions between Que and RBP occurred, the RBP conformation was changed. The tryptophan residues of the RBP were exposed to the polar microenvironment [12]. On the other hand, the excited state energy of the tryptophan residue was absorbed, caused by the reaction between the phenolic hydroxyl group in the Que molecule and the tryptophan residue in the RBP [41].

Notably, the maximum fluorescence emission wavelength (λmax) of the RBP–Que complex increased in comparison with the RBP sample and showed a characteristic trend of red-shift followed by blue-shift with the increased Que concentration. The red-shift phenomenon may be the reason that the RBP structure was unfolded further as a result of the non-covalent interactions, resulting in more tryptophan residues buried in the hydrophilic solvent environment [42]. The blue-shift of the R-Q120 sample was due to the micro aggregation of the complexes [43]. In conclusion, the tertiary conformation of RBP was altered by the non-covalent interactions, inducing dynamic changes on the protein structure and affecting the local microenvironmental polarity of the tryptophan residues.

#### 3.2.4. Fluorescence Quenching Mechanism

The mechanisms of non-covalent interactions between RBP and Que can be explored by a fluorescence quenching experiment.

**Figure 4:**
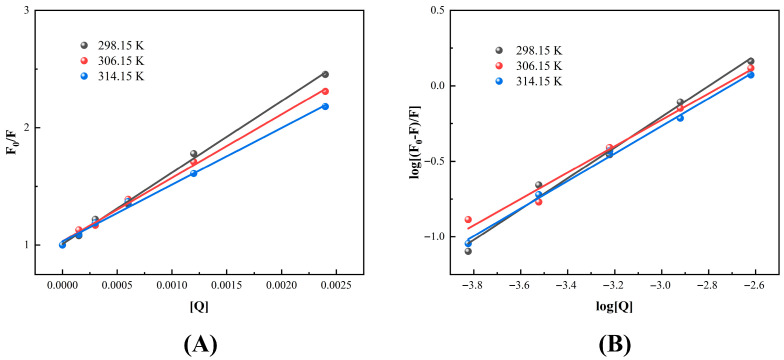
Stern–Volmer plots (**A**) and double logarithmic fitting plots (**B**) of rice bran protein (RBP) with the addition of quercetin (Que).

The fluorescence quenching data was analyzed by the Stern–Volmer Equation (8) and the Stern–Volmer plots are shown in Figure 4A:

(8) $$F_{0}/F=1+Ksv\left[Q\right]=1+Kq\tau_{0}[Q]$$

where F_0_—Fluorescence intensity of RBP without Que, F—Fluorescence intensity with Que, K_sv_—quenching constant, K_q_—Bimolecular quenching rate constant, τ_0_—Average lifetime of fluorophore, and [Q]—Que concentration.

The quenching constants, binding constants and binding sites of RBP–Que complexes are depicted in Table 1. The K_q_ was much higher than the maximum limitation value 2.0 × 10^10^ L·mol^−1^·s^−1^ for dynamic quenching. More importantly, the K_sv_ values were observed to decrease as the temperature increased. This was highly in accordance with the characteristics of static quenching [39]. The non-covalent forces which maintained the stability of the complexes were weakened by the temperature increasing, leading to the dissociation of the ground-state complexes and a decrease in the value of K_sv_. This finding was consistent with the study of Wang et al. [14] on the interactions between hemp seed protein and tannic acid, the mechanism of quenching was similarly determined to be static quenching.

Furthermore, the binding constants and the number of binding sites were obtained by fitting the quenching data with a double logarithmic Equation (9), and double logarithmic fitting plots are shown in Figure 4B:

(9) $$log[(F_{0}-F)/F]=logK_{a}+n\;log[Q]$$

where K_a_—binding constant and n—the average number of Que binding sites on each RBP molecule.

The fitting results showed that the K_a_ of the Que and the RBP were 713 L·mol^−1^, 247 L·mol^−1^, and 293 L·mol^−1^ in the experimental temperature range. It was noteworthy that the K_a_ values did not show a clear trend of regularity with increasing temperature, and the range of the values suggested that the binding interactions between the RBP and the Que were not particularly tight. Critically, the calculated number n was close to 1. The results demonstrated a 1:1 stoichiometric complex formation between Que and RBP. This was consistent with the study of non-covalent interactions between soy protein and chlorogenic acid [13].

The thermodynamic parameters of the binding process can be calculated by analyzing the binding constants at different temperatures using the Van ’t Hoff Equations (10) and (11). The thermodynamic parameters of the RBP–Que complexes are shown in Table 2.

(10) $$lnK_{a}=-∆H/\left(RT\right)+∆S/R$$

(11) $$∆G=∆H-T∆S=-RTlnK_{a}$$

where R—the gas constant, T—the absolute temperature, ΔG—the Gibbs free energy change, ΔH—the enthalpy change, and ΔS—the entropy change.

The calculations of the thermodynamic parameters showed that ΔH = −43.88 kJ·mol^−1^ and ΔS = −11.33 J·mol^−1^·K^−1^. ΔH < 0 indicated that the binding process was exothermic. ΔS < 0 indicated that the binding process led to an increase in the degree of ordering of the system (entropy reduction). ΔG < 0 indicated that the binding process was spontaneous. The negative ΔH and ΔS values observed confirmed thermodynamically that hydrogen bonding and van der Waals interactions dominated the RBP–Que complex formation, as is characteristic of enthalpy-driven binding processes [44]. The whole binding process was a spontaneous and exothermic enthalpy-driven reaction. Wang et al. [39] investigated the non-covalent interactions of soybean protein isolate with naringenin and the results were consistent with this study.

### 3.3. Functional Characteristics

#### 3.3.1. Solubility

**Figure 5:**
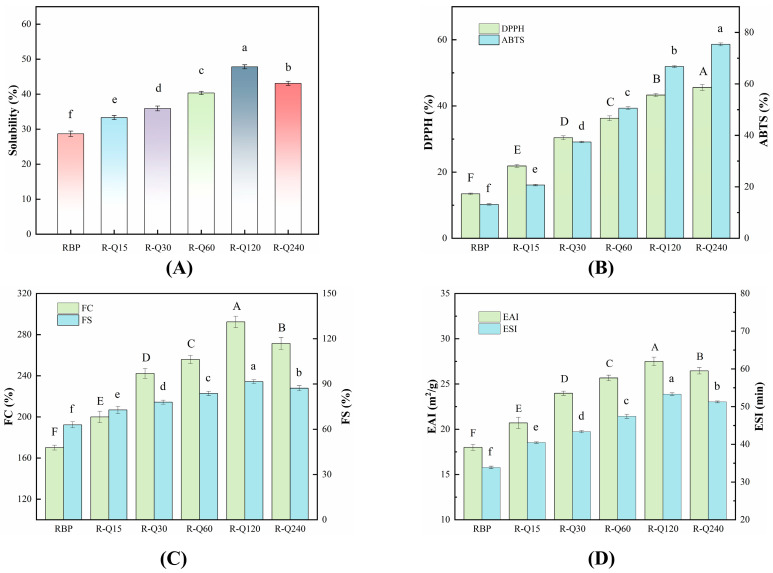
Solubility (**A**), antioxidant activities (**B**), foaming capacity (FC) and foaming stability (FS) (**C**), and emulsifying activity index (EAI) and emulsifying stability index (ESI) (**D**) of rice bran protein (RBP) and RBP–Quercetin (Que) non-covalent complexes. R-Q15, R-Q30, R-Q60, R-Q120, and R-Q240 represent RBP–Que complexes with different Que concentrations (15, 30, 60, 120, and 240 μmol/g protein, respectively). The error bars indicate the standard deviation (±SD) obtained from triplicate determinations (*n* = 3). Uppercase (A–F) indicate significant differences in DPPH, foaming capacity (FC) and emulsifying activity index (EAI) of RBP and RBP–Que complexes; lowercase (a–f) letters indicate significant differences in solubility, ABTS, foaming stability (FS) and emulsifying stability index (ESI) of RBP and RBP–Que complexes, respectively (*p* < 0.05).

Solubility is an important prerequisite for the effective application of proteins in the food processing industry. The solubility changes of the RBP–Que complexes are depicted in Figure 5A. The solubility of the complex increased significantly (*p* < 0.05) compared with the RBP. The solubilities of the RBP–Que complexes increased at the Que concentration 0–120 μmol/g, and the solubility of the R-Q120 sample reached the maximum (47.82%), which increased by 66.62% compared with that of the RBP (28.70%). The Que molecules were bound to the hydrophobic groups of the RBP, which made the hydrophobic groups were partially shielded, thus reducing the tendency of hydrophobic aggregation between protein molecules [45]. Furthermore, the hydrogen bonds formed by the phenolic hydroxyl groups of Que and the amino acid residues of RBP could also increase the hydrophilicity of the RBP molecules. In addition, the enhanced surface charge density promoted interprotein electrostatic repulsion, thereby effectively suppressing protein aggregation phenomena. The reduced particle size also contributed to the increased solubility. The solubility of the R-Q240 sample decreased slightly, which was due to the formation of a large number of insoluble aggregates [18]. Meanwhile, the enhanced hydrophobicity of the R-Q240 sample could also decrease the solubility. The experimental results of this study were in accordance with Wang et al. [12].

#### 3.3.2. Antioxidant Activities

The antioxidant capacities of RBP–Que complexes are depicted in Figure 5B. It was found that the DPPH and ABTS of RBP could be improved by the Que binding to the RBP. Compared to the RBP, the DPPH and ABTS radical scavenging rates of the RBP–Que complexes increased significantly (*p* < 0.05). This indicated that the effective loading of Que was useful to increase the antioxidant activities of RBP. The Que was rich in phenolic hydroxyl groups, which were able to quench free radicals (DPPH/ABTS) directly; thus, the antioxidant capacities of the RBP–Que complexes were enhanced. The preparations of the complexes were carried out under inert conditions; as we know, this was able to retain the activity of phenolic hydroxyl group to the maximum extent [17].

The active phenolic hydroxyl groups of Que were bound to the RBP structure through non-covalent interactions, which could contribute to the improvements in the antioxidant capacity of the complex, rather than simply physically mixing them together [10]. The non-covalent binding between the RBP and the Que could effectively improve the antioxidant property of RBP. Yin et al. [46] conducted a study on the reactions between apple polyphenols and four types of proteins. Their findings showed that apple polyphenols could significantly enhance the antioxidant properties of these proteins, which was consistent with our research findings.

#### 3.3.3. Foaming Properties

Foaming performance is a key functional indicator to evaluate the potential applications of proteins in food systems, which can reflect the ability of proteins to form interfacial membranes on air-water interface [47]. The foaming properties of the RBP–Que non-covalent complexes were investigated, and the experimental results are shown in Figure 5C. The FC and FS of the RBP–Que complexes were significantly (*p* < 0.05) higher than those of the RBP. The FC and FS of the R-Q120 sample reached 292.40% and 91.55%, which were 71.88% and 45.50% higher than those of the RBP, respectively. This improvement in foaming performance was mainly due to the structural changes of the RBP as a result of the addition of Que.

The foaming performance of proteins is closely related to their solubility, surface activity, and molecular flexibility; these can affect the distribution capacity of protein molecules on the air–water interface, the adsorption rate and the stability of the bubble system [48]. In this research, it has been found that the solubility and the flexibility of the RBP increased as a result of the non-covalent interactions. These enabled the RBP to arrange more rapidly on the interface, enhancing the foaming properties effectively [49]. However, excessive addition of Que may cause aggregation of protein molecules, disrupting the continuity of the air–water interfacial membrane and thus reducing the FC and FS [50]. This was in line with the observations of Hao et al. [26].

#### 3.3.4. Emulsifying Properties

The EAI and ESI are the core parameters to assess the emulsifying performance of proteins. The EAI is used to reflect the ability of protein to adsorb on the oil–water interface and reduce the interfacial tension, while the ESI is used to characterize the persistence of the emulsion in resisting the phases separation and maintaining the dispersed state. The EAI and ESI reveal the interfacial behavior and stability of proteins in the emulsion system separately [26]. As the natural emulsifier in the food system, protein forms a viscoelastic membrane through interfacial adsorption. Higher EAI and ESI values indicate the protein can form a better interface.

The emulsifying properties of RBP–Que complexes are shown in Figure 5D. It can be seen that the emulsifying properties of the RBP were enhanced effectively by the interactions between the Que and the RBP. The EAI and ESI of the complexes were significantly (*p* < 0.05) higher than those of the RBP. The EAI and ESI of the R-Q120 sample were 27.49 m^2^/g and 53.31 min, which were increased by 52.72% and 57.40%, respectively, compared with the RBP. The reasons were that the RBP structure was unfolded and the protein flexibility was increased by the non-covalent interactions. Flexible proteins adsorb and spread more easily on the oil–water interface [20]. In general, the decrease in particle size, the increase in the absolute value of ζ-potential, and the decrease in the number of hydrophobic groups can improve the solubility of protein. The increased solubility is useful to enhance the emulsifying properties [51]. These findings were consistent with the corresponding experimental results in this study.

## 4. Conclusions

In this study, the mechanisms of non-covalent interactions between RBP and Que were investigated and the functional properties were explored in further depth. The RBP–Que complex was formed. The non-covalent binding method can improve the functionality of rice bran protein. It was found that the complexes had more uniform particle size and a larger absolute value of potential compared with the RBP. The structure of the RBP was changed by the interactions. The result of fluorescence quenching confirmed that the Que was bound to the RBP mainly through hydrogen bonding and van der Waals forces, and the whole binding process was a spontaneous and exothermic process. The RBP–Que complex exhibited significantly better functional properties than RBP, with improved antioxidant, foaming and emulsifying properties. This study provides a new strategy for the application of RBP in food field. Future studies will delve into the application of RBP–Que complexes in a stabilized emulsion system.

## Figures and Tables

**Table 1:** The quenching constants, binding constants and binding sites of rice bran protein (RBP)–quercetin (Que) non-covalent complexes.

T (K)	K_SV_ (10^3^ L mol^−1^)	K_q_ (10^11^ L mol^−1^ s^−1^)	R^2^	K_a_ (10^2^ L mol^−1^)	n	R^2^
298.15	0.61 ± 0.02	0.61 ± 0.02	0.9988	7.13 ± 0.02	1.02 ± 0.06	0.9945
306.15	0.54 ± 0.02	0.54 ± 0.02	0.9982	2.47 ± 0.02	0.87 ± 0.07	0.9919
314.15	0.48 ± 0.02	0.48 ± 0.02	0.9980	2.93 ± 0.01	0.91 ± 0.03	0.9979

**Table 2:** The thermodynamic parameters of rice bran protein (RBP)–quercetin (Que) non-covalent complexes.

T (K)	ΔH (kJ mol^−1^)	ΔS (J mol^−1^ K^−1^)	ΔG (kJ mol^−1^)
298.15	−43.88	−11.33	−16.28
306.15			−14.02
314.15			−14.83

## Data Availability

The original contributions presented in the study are included in the article; further inquiries can be directed to the corresponding author.

## Abbreviations

The following abbreviations are used in this manuscript:

RBP	Rice Bran Protein
Que	Quercetin
ANS	8-Anilino-1-naphthalenesulfonic acid
SDS	sodium dodecyl sulfate
DPPH	1,1-diphenyl-2-picrylhydrazyl
ABTS	2,2′-azino-bis (3-ethylbenzothiazoline-6-sulfonic acid)
PDI	Polydispersity Index
FTIR	Fourier Transform Infrared
H_0_	Surface Hydrophobicity
FC	Foaming Capacity
FS	Foaming Stability
EAI	Emulsifying Activity Index
ESI	Emulsifying Stability Index
